# Study protocol: targeted delivery of interleukin-12 in combination with hepatic artery infusion pump therapy for patients with adrenocortical carcinoma liver metastases

**DOI:** 10.1186/s12885-026-16425-0

**Published:** 2026-06-27

**Authors:** Lindsay R. Friedman, Emily C. Smith, A. Leila Sarvestani, Alyssa V. Eade, Jason Ho, Tracey Pu, Carolina Larrain, Cathleen E. Hannah, Tamika Magee, Kathleen M. Smith, Audra A. Satterwhite, Sophia Xiao, Justine F. Burke, Surajit Sinha, Priyanka P. Desai, Kirsten Remmert, Michael J. Cavnar, James L. Gulley, Jeffrey Schlom, Jaydira del Rivero, Jonathan M. Hernandez

**Affiliations:** 1 Surgical Oncology Program, Center for Cancer Research, National Cancer Institute, National Institutes of Health, Bethesda, MD USA; 2 Developmental Therapeutics Branch, Center for Cancer Research, National Cancer Institute, National Institutes of Health, Bethesda, MD USA; 3 Complex General Surgical Oncology, Department of Surgery, University of Kentucky Markey Cancer Center, Lexington, KY USA; 4 Genitourinary Malignancies Branch, Center for Cancer Research, National Cancer Institute, National Institutes of Health, Bethesda, MD USA; 5 Center for Immuno-Oncology, Center for Cancer Research, National Cancer Institute, National Institutes of Health, Bethesda, MD USA

**Keywords:** Clinical trials, Adrenocortical carcinoma, Regional therapy, Liver cancer, Hepatic arterial infusion chemotherapy, Metastatic liver cancer, Immunotherapy, Interleukin-12, Surgery

## Abstract

**Background:**

Adrenocortical carcinoma (ACC) is a rare, aggressive malignancy often detected at an advanced stage with limited effective treatment options beyond first-line chemotherapy. The liver is a common site of metastasis, and patients frequently present with substantial hepatic disease burden that precludes complete resection. Locoregional therapies, including hepatic artery infusion pump (HAIP) delivery of floxuridine, have not been thoroughly evaluated for treatment of ACC liver metastasis, likely due to its rare incidence and highly aggressive nature. PDS01ADC (previously referred to as NHS-IL12) is a novel antibody-drug conjugate designed to deliver the immunomodulatory cytokine interleukin-12 to areas of tumor necrosis and modulate the tumor microenvironment to support immune surveillance and cytotoxic tumor responses. Combination of PDS01ADC with HAIP therapy (HAIP-delivered floxuridine plus systemic chemotherapy) was recently found to be feasible for clinical evaluation by an interim analysis in patients with colorectal liver metastases. Patients who received PDS01ADC with HAIP therapy had extended overall survival compared to patients who received HAIP therapy alone in a nonrandomized prior sequential study. We discuss herein the rationale and initiation of a new clinical trial arm to evaluate HAIP therapy with PDS01ADC for patients with ACC liver metastasis who have failed standard of care therapy.

**Methods:**

Patients with unresectable ACC liver metastasis and either resectable or no progressive extrahepatic disease will receive combination therapy including HAIP floxuridine and subcutaneous injection of PDS01ADC administered concurrently with systemic gemcitabine and oxaliplatin in 28-day cycles. The primary outcome measured is overall response rate as measured by Response Evaluation Criteria in Solid Tumors (RECIST v1.1) criteria. Secondary outcomes measured in this study include intrahepatic and extrahepatic progression-free survival, overall survival, and safety of PDS01ADC combination therapy with HAIP-delivered floxuridine.

**Discussion:**

For select patients with ACC liver metastasis, HAIP therapy with PDS01ADC may improve disease control and thereby prolong survival.

**Trial registration:**

Study ID NCT05286814 version 2026-05-04; https://clinicaltrials.gov/study/NCT05286814.

**Supplementary Information:**

The online version contains supplementary material available at https://doi.org/10.1186/s12885-026-16425-0.

## Plain language summary

Adrenocortical carcinoma (ACC) is a highly lethal malignancy with few treatment options, especially in the setting of distant metastases. The liver is a common site of disease spread, with numerous metastases frequently limiting locoregional therapy options that specifically target the liver. This study protocol describes the rationale for a new clinical trial arm for patients with ACC liver metastasis, evaluating high dose chemotherapy (HAIP floxuridine) delivered directly to the liver in combination with a targeted immunotherapy (PDS01ADC) designed to enhance immune responses within the tumor microenvironment. Combined with systemic chemotherapy (gemcitabine plus oxaliplatin), this approach leverages both liver-targeted and systemic (whole body) mechanisms to overcome treatment resistance and reduce tumor burden. The rationale to test this therapy for patients with ACC liver metastasis includes promising case studies using HAIP floxuridine for ACC liver metastasis as well as recent clinical data evaluating the safety and early efficacy of HAIP floxuridine combined with PDS01ADC in patients with colorectal liver metastases. It is important to expand this treatment evaluation to patients with ACC liver metastasis as this disease is highly aggressive with few options for effective treatment.

**Supplementary Information:**

The online version contains supplementary material available at https://doi.org/10.1186/s12885-026-16425-0.

## Introduction

Adrenocortical carcinoma (ACC) is a rare tumor with 0.5 to 2 new cases per million people per year globally that carries an exceedingly poor prognosis [1–4]. Average survival from time of diagnosis is approximately 12–15 months [5–7] with a 5-year mortality rate of 75–95% [6] secondary to metastatic disease progression [1, 2]. Of those patients that present with Stage IV disease, 33% will have synchronous liver metastasis [1, 8]. Moreover, up to 80% of patients who undergo primary tumor resection will subsequently develop locoregional (30–80%) [9] or distant metastasis (40–57%) [10, 11], with the most common sites being the liver (40–90%), lung (40–80%), or bone (5–20%) [5, 12–15]. The current accepted systemic regimens include etoposide, doxorubicin, and cisplatin plus mitotane (EDP-M) and streptosozin plus mitotane (S-M) [7, 16]. While EDP-M demonstrated a superior objective response to S-M, there was no significant difference in overall survival (14.8 months vs. 12 months, *p* = 0.07) when compared in an international phase III trial [6]. The high rates of recurrence and metastasis combined with low overall survival rates underscore the urgent need for alternative treatment strategies following first-line chemotherapy.

While there is a clear need for improved systemic treatment regimens, it must be noted that prolonged disease-free and overall survival have been reported following locoregional (i.e. resection or ablation) management of recurrent and metastatic disease, including the liver [8, 14, 17–19]. Control of hepatic disease may confer a survival advantage (22.8–76.1 months) in highly selected patient populations with favorable biology [9, 17, 20] as liver metastases have increased growth kinetics compared to other sites of metastases, with a volumetric doubling time of 27 days, compared to pulmonary (90 days) and nodal (95 days) sites as measured by computed tomography (CT) evaluation [20]. Given the aggressiveness of ACC liver metastasis, liver-directed strategies have been explored as a means to control disease and thereby prolong survival. Radiofrequency ablation and microwave ablation have demonstrated success in controlling liver metastasis; however, efficacy seems to be limited to small tumors (< 3 cm) that are not in close proximity to hepatic vasculature [18, 21]. Transarterial embolization offers targeted treatment by restricting blood flow to treat hepatic metastasis, though typically reserved for palliative intervention [22]. Selective internal radiation therapy including Yttrium-90 (Y90) radioembolization may be advantageous for patients with large multifocal disease; however, only few cases have been reported in literature [23–25].

**Fig. 1:**
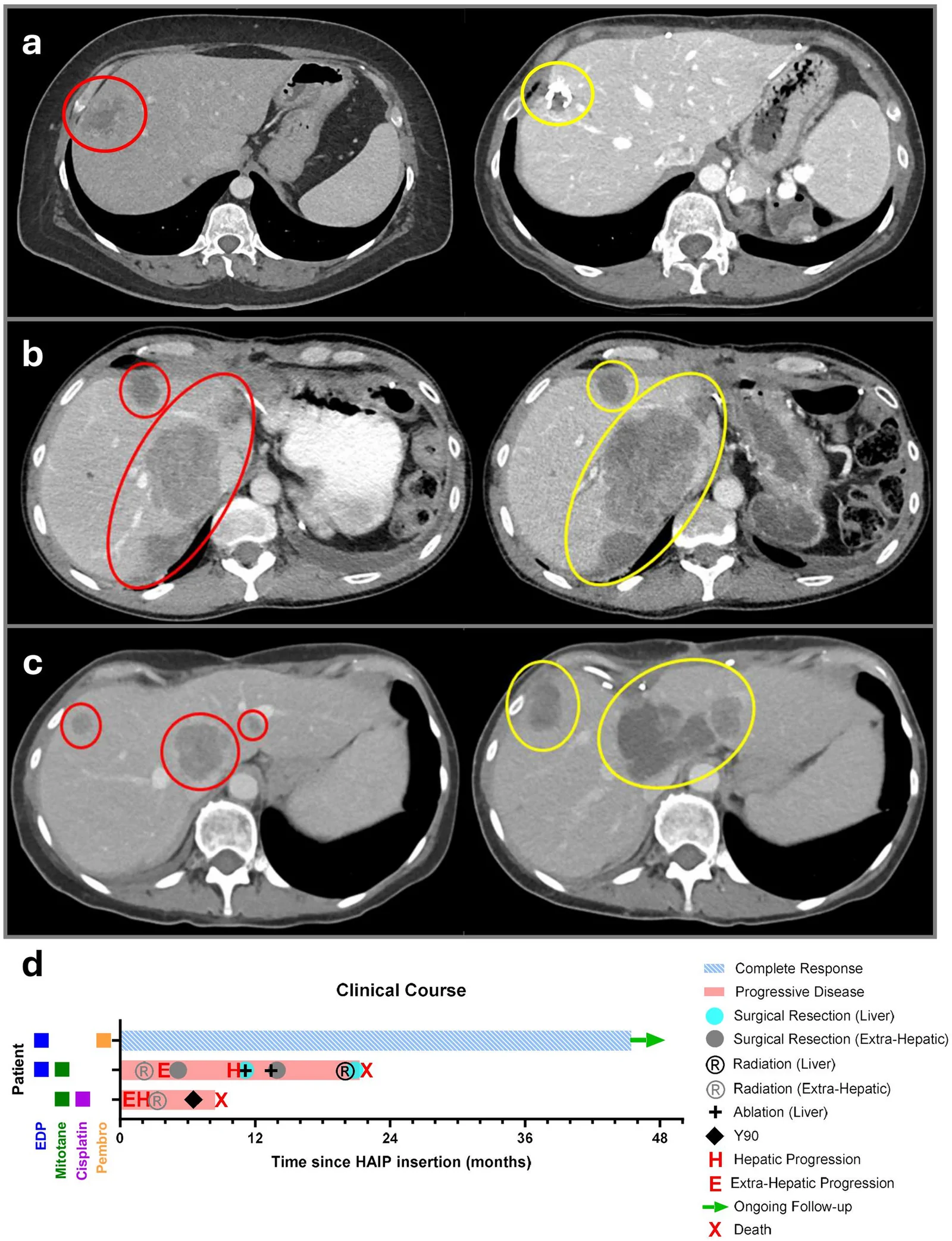
Patient Responses to HAIP-delivered Floxuridine for Locoregional Treatment ACC Liver Metastasis. (**a**) Axial CT comparing pretreatment heterogenous hepatic metastasis (red circle, left) compared to posttreatment expected post-surgical changes and clips within the liver (yellow circle, right) in patient with no evidence of recurrence at 44 months. (**b**) Axial CT demonstrating post-HAIP-treatment hepatic disease progression at 19 months (red circles, left) and 20 months (yellow circles, right) in a patient with progressive disease after transient control. (**c**) Axial CT comparing pretreatment hepatic disease (red circles, left) and post treatment hepatic disease progression at 2 months (yellow circles, right) in a patient with progressive disease. (**d**) Swimmer plots demonstrating patients’ clinical courses including disease response, progression, and both liver and extra-hepatic interventions. Top, middle, and bottom lines correlate with patient scans in (**a**), (**b**), and (**c**) respectively.

Hepatic artery infusion pump (HAIP) therapy is another locoregional approach to the management of metastatic liver disease [26–28]. HAIP therapy is facilitated by the dual blood supply of the liver, with hepatic tumor lesions preferentially perfused by the hepatic artery [29–31] allowing high-dose floxuridine administration that limits systemic exposure due to highly efficient first-pass metabolism [32]. Intriguingly, while HAIP therapy has not been systematically investigated in ACC liver metastasis, regimens containing 5-fluorouracil (5FU) have shown clinical activity in this disease [33]. Importantly, we previously reported two patients with ACC liver metastases who were managed with HAIP-delivered floxuridine, a precursor to 5FU [34]. Follow-up on these patients as of the data cutoff date (Jan 20, 2026) revealed one patient who maintains no clear evidence of disease 3.8 years after therapy (imaging shown in Fig. 1. a). The second patient experienced hepatic control of disease for approximately 10 months before demonstrating progression on follow-up imaging (shown in Fig. 1. b). A third patient treated after these initial two patients demonstrated immediate progression despite HAIP therapy (imaging shown in Fig. 1. c). These varied outcomes associated with the use of HAIP therapy for ACC liver metastases (summarized in Fig. 1. d) parallel observations seen in similar cohorts of patients with colorectal liver metastases and intrahepatic cholangiocarcinoma, highlighting the need for further study to optimize efficacy. Optimization of HAIP therapy for ACC may include refining patient selection across diverse patient populations and combining with complementary treatment strategies to maximize disease control.

Checkpoint inhibition has demonstrated promising yet transient responses in a subset of patients with metastatic ACC [35], suggesting underlying sensitivity to immunomodulation despite a typically immunosuppressive tumor microenvironment. Additional mechanisms of immunomodulation beyond checkpoint inhibition may be important to prime the tumor microenvironment, increase interactions between immune cells and tumor, and tune immune cells toward cytotoxic rather than immunosuppressive activities. Interleukin-12 (IL-12) is a cytokine that stimulates cytotoxic immune effectors (natural killer cells and T cell subsets) in addition to inducing differentiation of T cells to promote cell-mediated immunity and anti-tumor activity [36, 37]. Intratumoral IL-12 has been shown to mediate CD8 + T-cell tumor infiltration and multiple pre-clinical models replicate anti-tumor effects of recombinant human IL-12 (rhIL-12) [36, 38]. Unfortunately, systemic rhIL-12 was poorly tolerated due to severe systemic toxicity [39]. This led to the development of NHS-IL12 (now referred to as PDS01ADC), a novel immunocytokine composed of two IL-12 heterodimers fused to NHS76 human IgG1 antibody [40]. Specificity conferred by NHS76, an antibody that targets DNA accessible through compromised membranes of dead and dying cells, facilitates delivery of IL-12 within necrotic tumors to promote anti-tumor cell mediated immunity and cytotoxicity [41]. Combining targeted immunomodulation by PDS01ADC with high-dose liver-directed HAIP floxuridine and systemic chemotherapy to maximize tumor cell death, enhancing PDS01ADC accumulation in responding lesions, may confer significant benefit to patients with treatment-resistant metastases such that occur in the liver with ACC. An interim analysis of patients with microsatellite-stable colorectal liver metastases (in arm 1 of this trial) showed that addition of PDS01ADC to HAIP therapy was feasible without reducing delivery of floxuridine, and 7/9 patients had partial or complete responses at 6 months [42]. Notably, median overall survival of patients treated with PDS01ADC + HAIP therapy was significantly increased in comparison to that of patients treated with HAIP therapy alone in nonrandomized sequential studies. In context of these highly promising initial data for treatment of colorectal liver metastases, together with the case of studies of HAIP-delivered floxuridine for treatment of ACC liver metastasis, HAIP combined with PDS01ADC is worthy of investigation for this patient population with limited remaining treatment options.

## Methods

### Clinical trial design

**Fig. 2:**
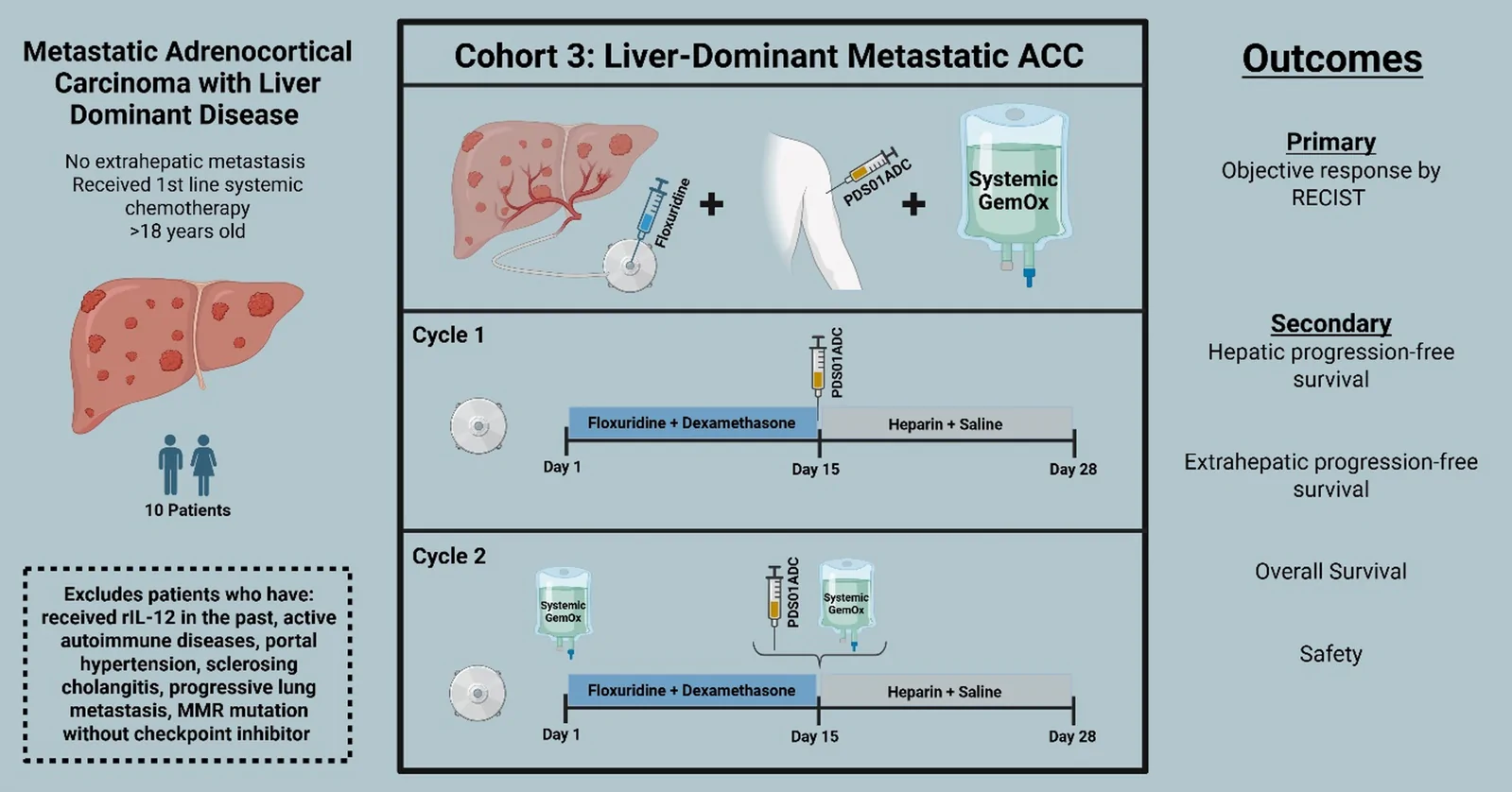
Trial Protocol Summary for NCT05286814 Cohort 3. Patients with metastatic adrenocortical carcinoma with liver dominant disease will be enrolled and receive surgical placement of a hepatic artery infusion pump. Patients will proceed to receive combination HAIP liver-directed floxuridine and dexamethasone, subcutaneous injections of PDS01ADC, and systemic gemcitabine and oxaliplatin as depicted. Outcomes measured will be objective response rate, hepatic and extra-hepatic progression-free survival, overall survival, and safety of combination therapy. ACC: adrenocortical carcinoma. MMR: mismatch repair. GemOx: gemcitabine + oxaliplatin. HAIP: hepatic artery infusion pump. Created in BioRender. Ho, J. (2026) https://BioRender.com/g1jrjga

This open label, single center, non-randomized Phase II study represents an additional arm recently added to NCT05286814 and is reflected in the ClinicalTrials.gov registry. The study expanded to include ACC on November 22, 2024 and is actively recruiting participants. The original protocol including patients with colorectal liver metastases (Cohort 1) and intrahepatic cholangiocarcinoma (Cohort 2) has been published [43]. This section addresses methods specific to ACC liver metastases, Cohort 3 (Fig. 2). Patients with histologically or cytologically confirmed metastatic ACC with unresectable liver disease will be enrolled to determine the safety and efficacy of PDS01ADC in combination with HAIP floxuridine and systemic gemcitabine and oxaliplatin (GemOx, Fig. 2). Patients may have extrahepatic intra-abdominal disease that is amenable to complete extirpation at concomitant time of pump placement. Any pulmonary metastasis must demonstrate stability (by RECIST v1.1 criteria) for 3 months prior to study enrollment. All eligible and enrolled patients will undergo surgical HAIP placement concomitant with extirpation of all apparent resectable non-hepatic intra-abdominal disease. Following recovery from surgery, patients will be dosed with floxuridine and dexamethasone in heparin/saline at the start of the first cycle according to patient-specific floxuridine dosing for constant delivery over 14 days: 0.12 mg/kg ideal average body weight x 30mL/pump flow rate for floxuridine and 1 mg/day x pump volume/pump flow rate for dexamethasone. At day 15, the pump will be flushed and filled with heparin/saline for delivery through day 28. PDS01ADC will be administered by subcutaneous injection on day 15 of the first cycle at a dose of 12 mcg/kg, consistent with PDS01ADC administration for cohorts 1 and 2 of this trial and below the previously determined maximum tolerated dose of 16.8 mcg/kg [44]. Beginning in cycle 2 and continuing through subsequent cycles, systemic chemotherapy (GemOx) will be administered on days 1 and 15. Floxuridine will be dose-reduced in each subsequent cycle (beginning at cycle 2) according to patient liver function tests (LFTs) per standard of care for HAIP therapy, and PDS01ADC will be dose-reduced proportionally to floxuridine(Table 1). In the event of a therapy hold due to liver function test abnormalities, treatment will not resume until values have recovered to protocol-defined thresholds, defined as AST ≤ 3 times the reference value and alkaline phosphatase and total bilirubin ≤ 1.2 times the reference value. Upon resumption, floxuridine will be restarted at 25% of the last administered dose and PDS01ADC will be resumed at 4 µg/kg. Systemic chemotherapy will be dose-reduced per standard of care.

**Table 1:** Dose reduction protocol for floxuridine and PDS01ADC

	Reference Value	% FUDR dose	PDS01ADC Dose
AST (SGOT) (at pump emptying or day of planned retreatment, whichever is higher)	0 to < 2 x reference value	100%	8 mcg/kg
	2 to < 3 x reference value	80%	8 mcg/kg
	3 to < 4 x reference value	50%	4 mcg/kg
	≥ 4 x reference value	Hold	Hold
ALK PHOS (at pump emptying or day of planned retreatment, whichever is higher)	0 to < 1.2 x reference value	100%	8 mcg/kg
	1.2 to < 1.5 x reference value	50%	4 mcg/kg
	≥ 1.5 x reference value	Hold	Hold
TOT BILI (at pump emptying or day of planned retreatment, whichever is higher)	0 to < 1.2 x reference value	100%	8 mcg/kg
	1.2 to < 1.5 x reference value	50%	4 mcg/kg
	≥ 1.5 x reference value	Hold	Hold

LFTs, including aspartate aminotransferase (AST), alkaline phosphatase (ALK PHOS), andtotal bilirubin (TOT BILI), will be obtained prior to the start of each treatment cycle. Dose reductions will be implemented according to the degree of LFT abnormality and the corresponding dose-modification criteria outlined in the table

Patients will be monitored for adverse events throughout treatment through clinical assessment, laboratory testing, and review of treatment-related symptoms prior to each cycle. Adverse event information will be collected including event description, date of onset, assessment of severity, relationship to the study intervention, and outcome. Events will be graded using the Common Terminology Criteria for Adverse Events version 5.0 (CTCAE v5.0). Adverse events will be managed according to protocol-defined criteria, including supportive care, additional evaluation, treatment delay, or dose modification. Criteria for discontinuation of PDS01ADC are as follows:

- Non-hematological, Grade 4 life threatening
- Grade 3 or Grade 4 inflammatory response syndrome (e.g., IRIS)
- Grade 3 or 4 interstitial pneumonitis
- NYHA Class III and IV congestive heart failure
- Grade 3 or 4 thrombocytopenia with a clinically significant bleeding requiring medical intervention
- Grade 4 hematologic toxicity lasting ≥ 7 days despite of medical intervention or Grade 4 neutropenia lasting > 5 days
- Cytokine release syndrome

### Eligibility

Eligibility criteria for cohort 3 ACC are consistent with criteria for cohorts 1 and 2 including that all participants must by 18 years of age or older, must have an Eastern Cooperative Oncology Group (ECOG) performance status of ≤ 1, and must have adequate organ and marrow function [43]. Eligible participants must have a histological diagnosis of ACC, also referred to as adrenocortical cancer, and have received at least one line of systemic chemotherapy.

### Exclusion

Exclusion criteria remain consistent with cohorts 1 and 2 as previously described [43] with the exception of exclusion criteria specific for ACC. Patients with incontrovertible radiographic evidence of additional abdominal disease outside of the liver (including the primary tumor) that is not amenable to complete surgical extirpation at the time of pump placement are excluded, as are patients with clinical evidence of portal hypertension (ascites, gastroesophageal varices, or portal vein thrombosis), diagnosis of sclerosing cholangitis, and patients with pulmonary metastases that have progressed by RECIST v1.1 criteria in the preceding 3 months prior to study enrollment. Furthermore, patients with known mismatch repair mutations who have not been treated with a checkpoint inhibitor are excluded.

### Study endpoints

The primary endpoint is objective response rate, defined as the proportion of patients with ACC who achieve a complete or partial response to treatment with PDS01ADC in combination with HAIP and systemic therapy. Patients will undergo radiographic assessment with CT scan every 8 weeks with an additional scan at 12 weeks and disease response will be determined using RECIST v1.1 criteria. Secondary endpoints include hepatic progression-free survival, extrahepatic progression-free survival, overall survival, and the safety and tolerability of the treatment regimen. (Fig. 2).

### Statistical analyses

The size of the ACC cohort was determined using a Simon optimal two-stage Phase II trial design to rule out an unacceptably low RECIST response rate (CR + PR) of 5% in favor of an improved response rate of 30%, with total accrual being 10 patients. The clinical response rate will be determined and reported along with a 95% confidence interval. Secondary endpoint probabilities will be determined using Kaplan-Meier estimates for all participants.

### Registration details and study timeline

This Phase II study is registered through clinicaltrials.gov (ID: NCT05286814) and sponsored by the National Cancer Institute (NCI). The study was expanded to include ACC on 11/22/2024 with an estimated completion date of 12/31/2028. This trial is actively recruiting participants. Patients will be followed for up to 5 years.

## Discussion

Despite significant advancement in systemic and targeted therapy for other solid malignancies, current chemotherapeutic regimens for ACC offer limited benefit with low response rates, especially in cases of liver metastases [2, 4, 15]. For those with metastatic ACC and significant liver disease burden, liver-directed HAIP therapy may offer durable benefit and control of disease [34]. Pre-clinical studies widely support the synergy of PDS01ADC with various cytotoxic agents able to induce cell death [45]. Furthermore, initial data for patients with microsatellite-stable colorectal liver metastases treated with PDS01ADC in combination with HAIP therapy are highly promising, with 78% of patients achieving a partial or complete response at 6 months and increased median overall survival in comparison to patients treated with HAIP therapy alone in a prior study by the same team. We will evaluate the role of combination therapy including HAIP floxuridine, subcutaneous injection of PDS01ADC, and systemic chemotherapy using gemcitabine and oxaliplatin, assessing overall response rates by RECIST v1.1 criteria as well as hepatic vs. extra-hepatic progression-free and overall survival in patients with unresectable ACC liver metastases. We will evaluate immune correlates of response by measuring serum cytokines and peripheral immune subsets at baseline through the first two cycles of therapy. Modulation of the intrahepatic tumor microenvironment will be evaluated through biopsies of liver metastasis following the first cycle.

## Supplementary Information

Supplementary Material 1.

## Data Availability

De-identified individual participant data from both the present article and the final study will be made available to qualified researchers beginning at the time of publication and up to 36 months after publication. Interested investigators should contact [Jonathan.Hernandez@nih.gov]. A data access agreement will be required prior to data release.
