# Supplementary material for: Study protocol: targeted delivery of interleukin-12 in combination with hepatic artery infusion pump therapy for patients with adrenocortical carcinoma liver metastases
Source: BMC Cancer. 2026 Jun 27;26:1099. doi: 10.1186/s12885-026-16425-0 (PMC13579813; doi:10.1186/s12885-026-16425-0)
Supplement: Supplementary file 1 [file 12885_2026_16425_MOESM1_ESM.docx]

**Supplemental Fig. S1. Surgical Extirpation and HAIP-delivered Floxuridine Demonstrate Persistent Locoregional Control of ACC with No Evidence of Disease at 3.75 Years Post-Treatment.** (a) Preoperative and pretreatment axial CT demonstrating large heterogenous, calcified primary ACC tumor (red circle). (b) Posttreatment axial CT demonstrating expected post-surgical changes at the primary site with no evidence of local recurrence of disease.
